# Central Nervous System Effects of the Second-Generation Antihistamines Marketed in Japan -Review of Inter-Drug Differences Using the Proportional Impairment Ratio (PIR)-

**DOI:** 10.1371/journal.pone.0114336

**Published:** 2014-12-12

**Authors:** Tatsuya Isomura, Takeshi Kono, Ian Hindmarch, Norimasa Kikuchi, Aya Murakami, Kyoko Inuzuka, Seiji Kawana

**Affiliations:** 1 CLINICAL STUDY SUPPORT, Inc., Nagoya, Aichi, Japan; 2 Institute of Medical Science, Tokyo Medical University, Tokyo, Japan; 3 Department of Dermatology, Nippon Medical School Chiba-Hokusoh Hospital, Chiba, Japan; 4 University of Surrey, Guildford, Surrey, United Kingdom; 5 Department of Dermatology, Nippon Medical School, Tokyo, Japan; Medical School of Hannover, Germany

## Abstract

**Background:**

Second-generation antihistamines (AHs) have, in general, fewer sedative effects than the first-generation. However, important inter-drug differences remain in the degree of cognitive and/or psychomotor impairment. The extent to which a particular compound causes disruption can be conveniently compared, to all other AHs, using the Proportional Impairment Ratio (PIR). Although the PIR can differentiate the relative impairment caused by individual drugs, there is no indication of the reliability of the ratios obtained.

**Objective:**

To calculate the PIRs –together with 95% confidence intervals (CIs), as an index of reliability– and compare AHs currently, or soon to be, available in Japan, with respect to their intrinsic capacity to cause impairment.

**Methods:**

Results from studies of cetirizine, desloratadine, ebastine, fexofenadine, levocetirizine, loratadine, mequitazine, and olopatadine were included in the PIR calculations. All data utilised came from crossover studies in healthy volunteers which were randomised and placebo and positive-internal controlled. Existing databases from studies reporting the sedative effects of AHs on objective (speed, accuracy, memory) and subjective (feeling) psychometrics were augmented, via results from suitable studies published after the previous reviews. The null value for a PIR was one.

**Results:**

A total of 45 studies were finally included for this review. Of the AHs assessed, fexofenadine, ebastine, and levocetirizine showed a PIR for objective tests of 0. However, only fexofenadine (PIR = 0.49) had an upper limit of the 95% CI of less than 1. Fexofenadine, levocetirizine, desloratadine, olopatadine, loratadine, and mequitazine all had a PIR for subjective ratings of 0, but the upper limits of the 95% CIs were all in excess of 1, although fexofenadine (PIR = 2.57) was the lowest.

**Conclusions:**

The results show that there are differences between second-generation AHs in the extent of sedation produced. However, subjective ratings indicate that patients may not necessarily be aware of this.

## Introduction

Antihistamines (AHs), especially newer oral AHs, are the most widely used therapeutic option to manage allergic diseases such as allergic rhinitis, urticaria, and other allergic skin disorders. In Japan, a number of the newer, so-called second-generation, AHs have been launched on the market, as in other countries. Although most of these medications still require a prescription, some of them have recently been made available over the counter.

The treatment effects of AHs are primarily due to antagonism of histamine-1 receptors (H_1_ receptors) in targeted tissues. AHs that cross the blood-brain barrier and bind to H_1_ receptors in the brain suppress central nervous system (CNS) arousal and disrupt circadian sleep-wake rhythmicity [1], thus impairing both cognitive function and psychomotor performance, including attention, memory, sensorimotor coordination, information processing, and psychomotor performance [2], [3]. Second-generation AHs are generally considered to have a lower potential for H_1_ receptor occupancy in the brain compared to the older, first-generation medications; however, there are differences among the second-generation drugs in the degree to which an administered drug passes through the blood-brain barrier and causes cognitive and psychomotor impairment.

In reviews of the literature [4], [5], the untoward effects of AHs were ranked using a proportional impairment ratio (PIR). The PIR is a calculation technique adapted from that used in pharmacovigilance [6] and shows whether the use of an AH is associated with psychomotor impairment and, if so, the extent of that impairment when compared to the effects of other AHs. The greater the PIR, the greater are the impairments associated with the use of that AH. In the previous reviews, the calculated PIRs demonstrated much greater psychometric impairment with first-generation than with second-generation AHs. However, not all second-generation drugs were found to be totally free from cognitive and psychomotor effects and there are inter-drug differences in the extent to which essential intellectual and behavioural activities are disturbed. In all previous reviews using the PIR [4], [5], the calculated value of PIR was a point estimate - a single number, without any indication of the reliability of the estimate. This point estimate does not differentiate between a more reliable PIR derived from 100 test results or that calculated from a database of only 10 test results.

In order to reduce the uncertainty inherent in comparing PIRs derived from databases of different sizes and especially when only a small number of test results are available, a 95% confidence interval (CI) was used. Interval estimates, such as 95% CIs, expand on point estimates by incorporating the uncertainty of the point estimates [6]. The larger the sample size of the test results, the narrower the CI around the PIR, i.e., the more reliable or precise the estimation.

The aim of the current review was to assess the cognitive and psychomotor function potential of second-generation AHs marketed in Japan using the PIRs and their 95% CIs. The data used for the PIR calculation were extracted from the studies previously reviewed by Shamsi & Hindmarch (2000) [4] and McDonald et al (2008) [5], together with those published after their reviews, if any.

## Methods

### Study selection

Relevant studies were selected from the existing database collated in the previous literature reviews [4], [5], as well as newly identified studies.

The drugs of interest in the current review were the second-generation AHs that were available or expected to be available in Japan. Therefore, from the existing database, studies of the following second-generation AHs were selected: cetirizine, desloratadine, ebastine, fexofenadine, levocetirizine, loratadine, mequitazine, and olopatadine. Not all of the second-generation AHs on the market were included, as some of the drugs, such as bepotastine and epinastine, did not appear in both of the previous reviews. Ketotifen, usually counted as a second-generation AH in Japan, was also not included. This was because: i) ketotifen was categorized as a first-generation drug in the previous reviews; and ii) very striking impairments, for instance, those caused by the first-generation AHs or ketotifen, would reduce the value of the PIRs for all other AHs, so that excluding such drugs was recommended [6]. Combination drugs were not of interest in this analysis. Terfenadine was not included as it was withdrawn from the market in Japan due to cardiotoxicity and is no longer available.

New studies were identified from a literature search of PubMed for papers published between March 2006 and June 2012, which provided a 2-year overlap of the search conducted by McDonald et al (2008) [5] to make sure that no study was missed. As in previous reviews [4], [5], the current review included randomized, placebo- and positive-internal controlled, crossover studies which reported the effects of AHs on cognitive function and psychomotor performance in healthy volunteers. The following search terms were included: 1) “antihistamine” or “H_1_ antagonist” or “psychomotor performance”; 2) “placebo”; and 3) the specific drug names mentioned above. Studies had to be psychopharmacological studies which assessed the CNS effects of the drugs, per se, and not the clinical effectiveness of the drug, and of particular interest were studies using standardized, quantitative methods for measuring any drug-induced changes on cognitive and psychomotor performance. Studies performed in children, i.e. under the age of 18 years, or patients, where the effects of disease could compromise the psychometrics, and those investigating the interaction with alcohol or other CNS drugs were excluded.

The psychometric tests used to evaluate cognitive and psychomotor performance were classified, as in previous reviews [4], [5], by grouping together those tests measuring similar CNS characteristics. As listed in Table 1, the measures were grouped into nine categories: eight “objective”, e.g. reaction time, tracking ability, etc., measures (codes: A-H) and one “subjective”, e.g. subjects' ratings of fatigue, sleepiness, etc., measure (code: I). The term ‘objective’ implies a psychometric where the subject's reaction and level of CNS arousal is quantified, that is, measured numerically, both as speed of reaction, number of items recalled, accuracy of mental arithmetic, etc. and from psycho-physiological assessments, e.g. electroencephalograph (EEG), actigraphy, etc., whereas ‘subjective’ refers to a subject's feeling of sedation, impairment, mood, etc.

**Table 1:** Categories of psychometric tests.

Code	Category	Test
A	Psychomotor Performance	brake reaction time
		actual car driving test
		car following test
		simulated car tracking task (SCTT)
		simulated accident avoidance
		simulated assembly line task (SALT)
		simulated driving reaction time
		standard deviation of lateral position
B	Psychomotor Speed	choice reaction time
		simple reaction time
		reaction tasks
C	Sensorimotor Co-ordination	continuous tracking task
		compensatory tracking task
		tracking task
		visuo-motor coordination
		pursuit rotor
		vigilance and tracking test
D	CNS Arousal, Information Processing	critical flicker fusion
		digit symbol substitution
		stroop word/color testing
		multi-attribute task battery (MAT)
		rapid visual information processing (RVIP)
		symbol digit coding
		matching paradigm
E	Memory	6-letter memory recall
		delayed memory recall
		digit memory recall
		short term memory
		Sternberg memory scanning task
		memory scanning task
F	Sensory Skills	aeromedical vigilance test
		divided attention test
		shifting attention test
		test of variables of attention (TOVA)
		sustained attention
		dynamic visual acuity
		visual discrimination time task (VDT)
		visual vigilance
G	Motor Ability	-
H	Physiological	body sway
		electroencephalograph (EEG) (sleep latency)
		multiple sleep latency test (MSLT)
		actigraphy
I	Subjective Ratings	addition research center inventory (ARCI-49)
		visual analogue scale
		global rating of performance
		global rating of sleepiness
		line analogue rating scale
		Samn-Perelli fatigue rating
		Stanford Sleepiness Scale
		adjective check list
		Bond and Lader's visual analogue scale

### Data extraction

For each drug at all doses tested, test results were classified as evidence of either “impairment” or “no impairment”. This classification was based on whether a statistically significant difference (p<0.05) was observed between the drug and placebo on each individual psychometric test. If there were multiple test points, results were counted as “impairment” when a significant worsening of test scores compared to placebo values was found at any of the post-treatment test points.

When results from a single trial were divided between different publications, they were counted as originating from one study. The scores from psychometric tests, where multiple outcome measures were presented in the results, were only counted as independent tasks when there was no possible interdependence, reciprocal relationship, or trade-off, e.g. speed-accuracy, with other measured outcomes from the same test. When there was evidence of contamination or interdependence of scores, only the result from the principal psychometric result was counted.

### Validity assessment of test results

The current review concentrated only on the results of studies where the verum (positive-internal control) successfully demonstrated - through a comparison with placebo - the sensitivity of the psychometric test(s) employed [7]–[10]. A test was deemed sensitive if a statistically significant (p<0.05) impairment of psychomotor/cognitive function was found at least at one time point during the course of the assessment.

### PIR calculation

**Figure 1:**
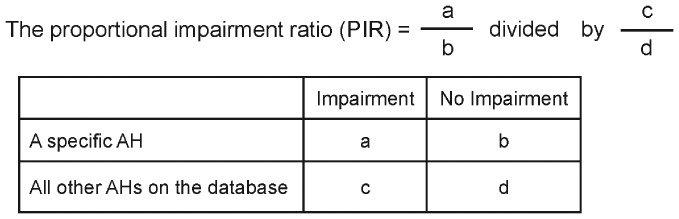
Proportional impairment ratio calculation formula for an antihistamine. a: Number of tests showing ‘impairment’ with the named antihistamine (AH). b: Number of tests showing ‘no impairment’ with the named AH. c: Number of tests showing ‘impairment’ with all other AHs. d: Number of tests showing ‘no impairment’ with all other AHs.

The PIR values were calculated for all AHs included in the current review. The formula for PIR calculation is shown in Fig. 1. The PIR is calculated for each AH and provides a proportionate index for the strength of impairment where the comparator is all other AHs included in the review. The null value for a PIR is 1, i.e. an indication there is no difference between the AH in question and all other AHs under consideration. PIR values behave in a similar fashion to the odds ratio (the lower the PIR, the less the strength of impairment against all other AHs) [6]. In addition to the PIR values, their 95% CIs were calculated using Cornfield's method, as an index of the reliability of the PIR [11].

The PIR calculations were performed separately for objective and subjective tests. Additionally, for the objective tests, the PIR and its associated 95% CI were also computed for each AH by dose. This is because, when CNS effects are compared between AHs, the dose of AH administered is a significant element to be considered, as the needs of clinical practice often determine that supra-recommended clinical doses are required to provide clinical efficacy [12].

All statistical calculations were performed using STATA version 9.0 software.

## Results

**Figure 2:**
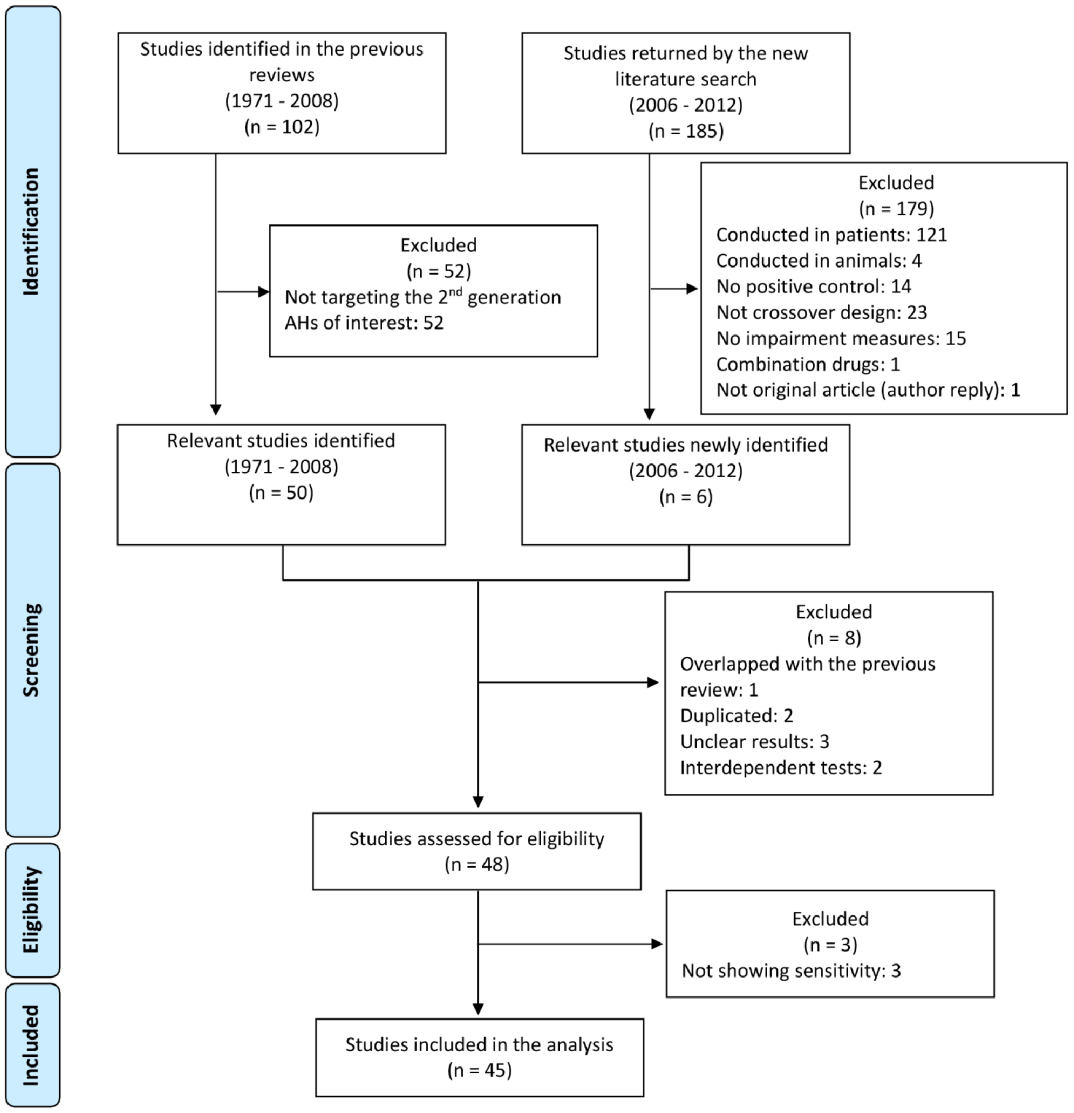
Flow diagram of study selection.

The flow diagram for the data extraction process is shown in Fig. 2. Of 102 studies identified in the previous reviews [4], [5], 50 studies relating to the second-generation AHs were selected as relevant to current Japanese usage. Although a new literature search returned 185 studies, the majority (121 studies) were conducted in patients. Six studies fully satisfied the selection criteria, afterwards.

Furthermore, a strict application of the present extraction criteria to studies identified led to 11 studies being omitted from current analysis: viz. 3 studies because the effects of the verum (positive-internal control) failed to demonstrate the sensitivity of the psychometrics to sedation and cognitive impairment; 1 study due to overlapping data; 2 studies because results were duplicated; 3 studies due to ambiguous/unclear results in the published paper; and 2 studies, Boyle et al (2006) [13] and Vermeeren and O'Hanlon (1998) [14], because of an interdependence of test results.

Boyle et al (2006) [13] analysed multiple outcome measures, and, in their discussion of results, recognised that some of the outcome measures were interrelated due to a speed/accuracy ‘trade-off’. Paradoxically, however, these interrelated scores were regarded as independent, discrete and separate measures in a later calculation of PIR by McDonald et al (2008) [5]. Furthermore, although included in McDonald et al (2008) [5], the paper by Vermeeren and O'Hanlon (1998) [14] was excluded from the current analysis - as it was from the original PIR paper by Shamsi and Hindmarch (2000) - for reasons of inconsistency in the data reported and the lack of clarity in the presentation of results used for the calculation of PIR values [15].

A total of 45 studies were finally used for the new PIR calculation. The list of studies and tests included in this review is presented in S1 Table [16]–[60].

**Figure 3:**
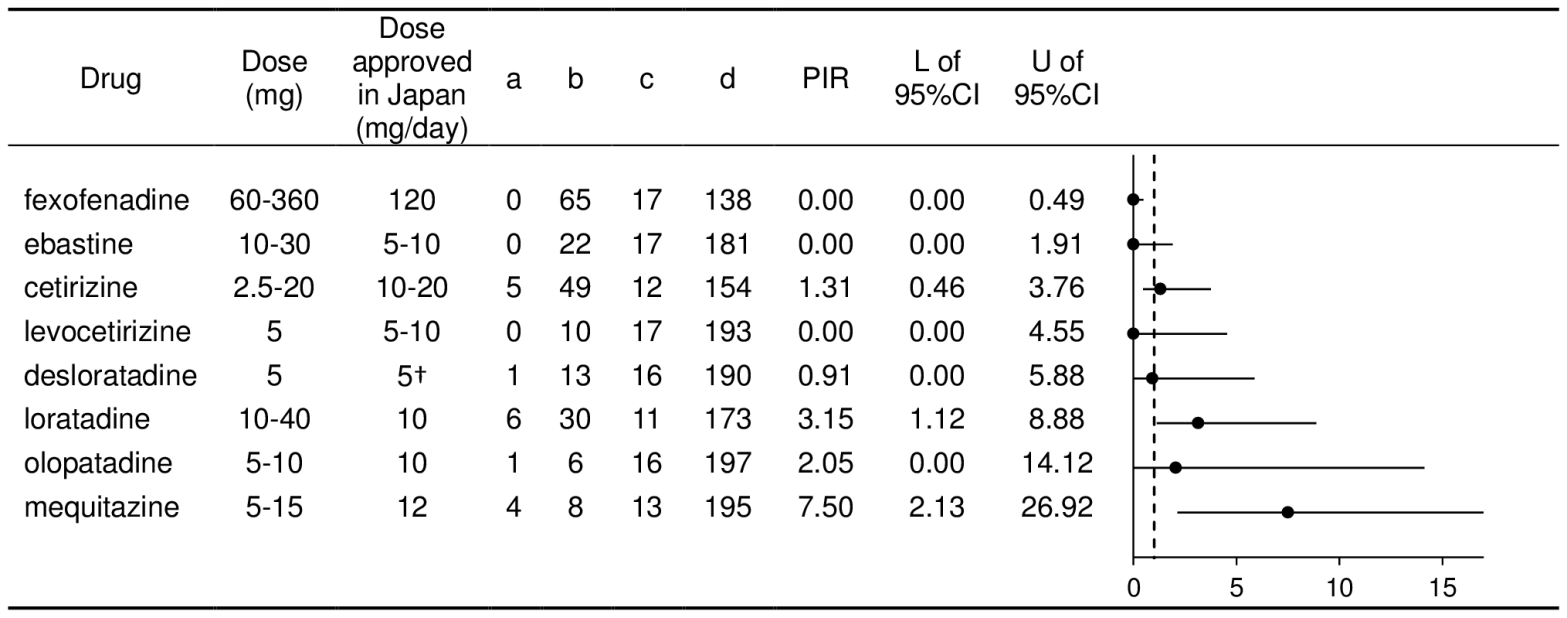
Calculation of proportional impairment ratio for objective tests for the second-generation antihistamines in Japan. a: Number of tests showing ‘impairment’ with the named antihistamine (AH). b: Number of tests showing ‘no impairment’ with the named AH. c: Number of tests showing ‘impairment’ with all other AHs. d: Number of tests showing ‘no impairment’ with all other AHs. L: Lower limit. U: Upper limit. The vertical dotted line in the figure of PIRs shows a value of 1.

**Figure 4:**
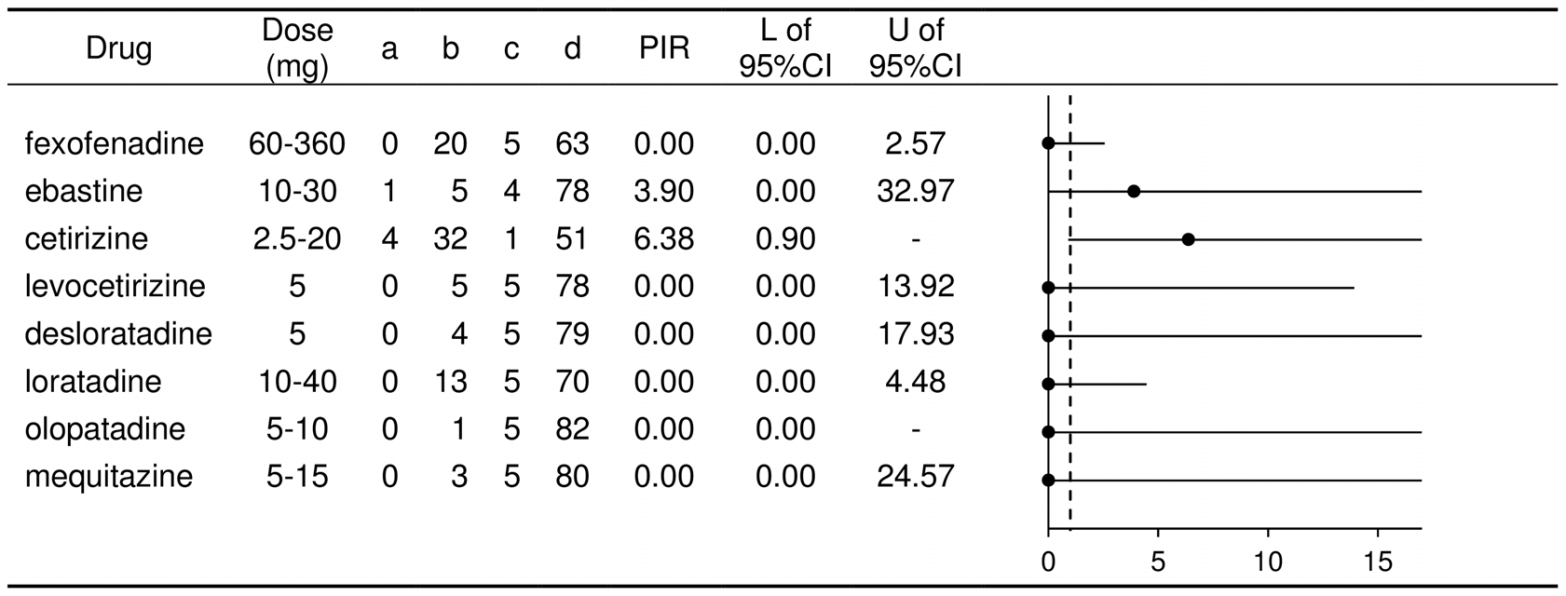
Calculation of proportional impairment ratio for subjective tests for the second-generation antihistamines in Japan. a: Number of tests showing ‘impairment’ with the named antihistamine (AH). b: Number of tests showing ‘no impairment’ with the named AH. c: Number of tests showing ‘impairment’ with all other AHs. d: Number of tests showing ‘no impairment’ with all other AHs. L: Lower limit. U: Upper limit. The vertical dotted line in the figure of PIRs shows a value of 1.

The number of test results detecting impairments and no impairments for each AH is summarized in Table 2. Using these data, PIRs were separately calculated for objective tests (PIR-O) and subjective tests (PIR-S), and the results are shown in Fig. 3 and Fig. 4, respectively. It should be noted that, since the study reported by Vacchiano et al (2008) [49] used cetirizine, one of the second-generation AHs, as a positive-internal control, the study results were included in the PIR calculation of all the other AHs except for cetirizine.

**Table 2:** Number of test results showing impairment and no impairment for each antihistamine (AH).

Drug	No. of tests showing impairment		No. of tests showing no impairment	
	Objective	Subjective	Objective	Subjective
cetirizine	5	4	49	32
desloratadine	1	0	13	4
ebastine	0	1	22	5
fexofenadine	0	0	65	20
levocetirizine	0	0	10	5
loratadine	6	0	30	13
mequitazine	4	0	8	3
olopatadine	1	0	6	1

Of the 8 AHs included in this review viz. cetirizine, desloratadine, ebastine, fexofenadine, levocetirizine, loratadine, mequitazine, and olopatadine, a PIR-O value of 0 was obtained for fexofenadine, ebastine, and levocetirizine. However, the estimated PIR and its 95% CI were less than 1 only for fexofenadine (0.49). The upper limits of the 95% CI for ebastine and levocetirizine were greater than 1 (1.91 and 4.55, respectively). Although the PIR-O for desloratadine was 0.91, the upper limit of its 95% CI was 5.88. Cetirizine and olopatadine had PIR-Os of 1.31 and 2.05, respectively. Loratadine and mequitazine had PIR-Os of 3.15 and 7.50, respectively, and even the lower limits of the 95% CIs were greater than 1 for both substances.

Fexofenadine, levocetirizine, desloratadine, olopatadine, loratadine, and mequitazine all had a PIR-S of 0. However, all of the upper limits of the 95% CIs were greater than 1. The upper limits of the 95% CI for levocetirizine, desloratadine, and mequitazine were 13.92, 17.93, and 24.57, respectively. Fexofenadine had the lowest upper limit for the 95% CI among the AHs (2.57). The PIR-Ss for ebastine and cetirizine (3.90 and 6.38) were greater than those observed for the PIR-Os (0.00 and 1.31).

**Figure 5:**
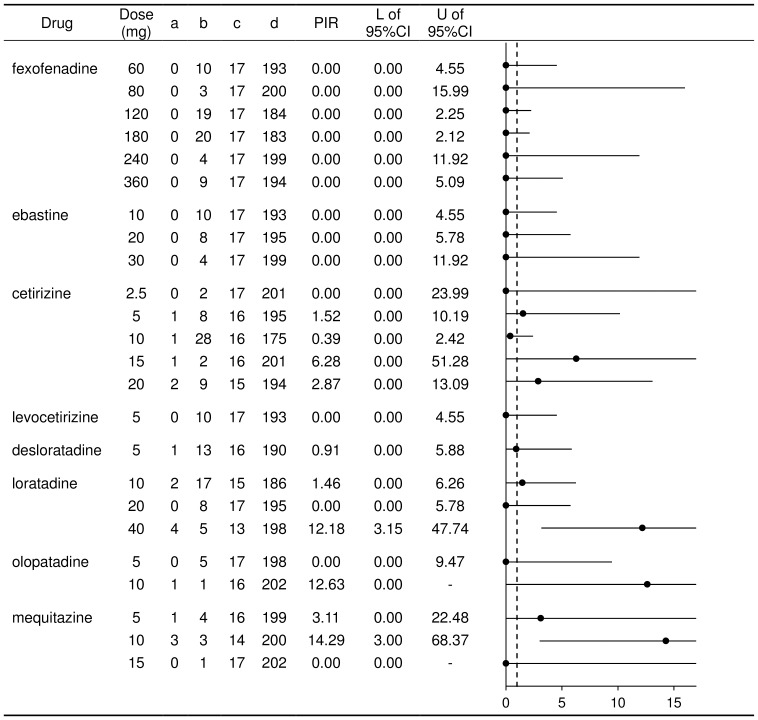
Calculation of proportional impairment ratio for objective tests by dose. a: Number of tests showing ‘impairment’ with the named antihistamine (AH). b: Number of tests showing ‘no impairment’ with the named AH. c: Number of tests showing ‘impairment’ with all other AHs. d: Number of tests showing ‘no impairment’ with all other AHs. L: Lower limit. U: Upper limit. The vertical dotted line in the figure of PIRs shows a value of 1.

The numbers of tests showing impairment and no impairment were tabulated for a range of doses (Table 3), and the PIR-O values were calculated (Fig. 5). For fexofenadine and ebastine, the PIR values were 0 at all doses. There was no drug at a specific dose having an upper limit of the 95% CI below 1. For cetirizine and loratadine, a difference in the PIR-O was demonstrated by dose. For levocetirizine, the only available information was with a dose of 5 mg. Similarly, the PIR-S values by dose were calculated, but it was not possible to evaluate the results because most of the 95% CIs were too wide due to lack of data for a range of doses.

**Table 3:** Number of test results showing impairment and no impairment for each AH by dose.

Drug	Dose(mg)	No. of Tests showing impairment		No. of Tests showing no impairment	
		Objective	Subjective	Objective	Subjective
cetirizine	2.5	0	0	2	1
	5	1	1	8	4
	10	1	2	28	20
	15	1	1	2	1
	20	2	0	9	6
desloratadine	5	1	0	13	4
ebastine	10	0	0	10	3
	20	0	0	8	2
	30	0	1	4	0
fexofenadine	60	0	0	10	2
	80	0	0	3	1
	120	0	0	19	7
	180	0	0	20	6
	240	0	0	4	2
	360	0	0	9	2
levocetirizine	5	0	0	10	5
loratadine	10	2	0	17	8
	20	0	0	8	2
	40	4	0	5	3
mequitazine	5	1	0	4	1
	10	3	0	3	1
	15	0	0	1	1
olopatadine	5	0	0	5	1
	10	1	0	1	0

## Discussion

In this review of major second-generation AHs marketed in Japan, data were selected from the reviews of Shamsi & Hindmarch (2000) [4] and McDonald et al (2008) [5], together with results from suitable new studies published subsequent to the last review. PIRs were recalculated for all drug dose regimens together with 95% CIs. The new PIRs and CIs enabled the rank comparison of all second-generation AHs that are available or expected to be available in Japan as regards their impact on cognitive and psychomotor functions. These rankings reflect the overall psychopharmacological impairment likely to be associated with the clinical use of a particular drug compared with the average of the class of drugs as a whole. The present results appear substantially consistent with the results from the previous reviews with regards to the results that AHs, such as fexofenadine, showed lower PIR values.

Cognitive and psychomotor impairment following the use of antihistamines is a consequence of the extent to which individual molecules penetrate the brain and act as inverse agonists at H_1_ receptor sites. The results of PIR-O and PIR-S suggest that fexofenadine be ranked as the least impairing AH compared to all other drugs assessed in this review. The 95% CI associated with the fexofenadine PIR reflects the reliability of the ranking. Although the zero and near zero PIR values for desloratadine and levocetirizine suggest that the drugs have a favourable ranking as regards cognitive and psychomotor impairment, this must be tempered by the lack of robust psychometric data from dose-ranging studies. The small database makes the PIR unreliable, as indicated in the large CI range. The PIR for olopatadine shows it to be ranked approximately 8 times more impairing than the average for all other second-generation AHs marketed in Japan. Notable differences were not demonstrated in PIR-S values; however, this may paradoxically suggest the important point that people tend not to be aware of cognitive impairment when their cognitive functions are actually impaired. The distinction between “subjective” and “objective” is very important, especially in clinical practice where patients may report feeling alert and unimpaired, while the AH prescribed to them is actually causing CNS sedation and thereby impairing cognitive and psychomotor function.

The Consensus Group on New Generation Antihistamines (CONGA) has proposed three essential criteria for defining a non-sedating AH: 1) there should be no “incidence of subjective sleepiness”; 2) there should be no impairment of “objective and psychomotor functions”; and 3) there should not be a significant amount of H_1_ receptor occupancy on positron emission tomography (PET) [12]. CONGA requires that these criteria are met at supra-clinical dose regimens, because some AHs show a rise in H_1_ receptor occupancy in a dose-dependent manner and because cognitive and psychomotor impairment are also dose-related. Furthermore, in clinical practice, patients are frequently prescribed AHs at higher dose regimens than those recommended by the manufacturer especially in those who do not respond satisfactorily to standard dose regimens. The absence of any dose-ranging studies excludes the classification of either desloratadine or levocetirizine as ‘non-sedative’ according to the CONGA criteria [12]. Although ebastine in the range of doses studied is seemingly as good as fexofenadine, the dose range studied is only 3 times that of a basic single dose, whereas it is 6 times with fexofenadine, and both loratadine and cetirizine were studied at 4 times a basic single dose. Loratadine and cetirizine showed dose-ranging impairment, which meant that neither would satisfy the CONGA requirements for classification as a non-impairing AH.

Impairment of CNS activities potentially results in accidents [7], [61], poor scholastic/academic achievement [62], reduced quality of life [63], and poor work productivity [64]. Any drug that induces impairment, even if it is mild, will cause certain illness-related impairments and raise the risk of accidents in safety-critical situations in the home, at work, or on the road.

It should be noted that PIRs are not, in themselves, objective assessments. PIRs represent the relative ranking of the effects on cognitive and psychomotor function of an individual antihistamine, compared to all other drugs in the same class. As such, there is a broad concordance between the results of those PIRs, shown to be reliable, and the percentage of H_1_ receptor occupancy in the brain on PET scans [65]. The percentage of occupancy varies among the different second-generation AHs, but all of the second-generation drugs show much lower percentages compared to the first-generation drugs. Fexofenadine had the lowest percentage, followed by ebastine, among the second-generation drugs of interest in the current review.

The impact of cognitive impairment varies between subjects. Therefore, the reliability of the PIR depends on homogeneous characteristics and condition of the subjects. To remove between-subject variation, as in previous PIR reviews [4], [5], the current analysis focused only on the studies using cross-over designs, where each subject served as his own control [66]. The PIR values calculated can be considered reliable to some extent.

Moreover, the PIRs can only reflect the results of data obtained from published papers and, as such, will be influenced by publication bias; for example, sponsors' decisions regarding which data to publish, statistically significant results with small sample sizes, i.e. inadequate statistical power, leading to false-negative conclusions, etc. It is clear, from Fig. 3, that there are limited data from fixed-dose studies of olopatadine, levocetirizine, an enantiomer of cetirizine, and desloratadine, a metabolite of loratadine. Furthermore, there are no published dose-ranging studies for any of these drugs, which make an evaluation of their potential for causing impairment impossible within the context of CONGA guidelines.

## Conclusions

In conclusion, the results suggest that there are noticeable inter-drug differences in the extent of the objectively determined sedative effects produced by second-generation AHs. However, the results from assessments of subjective ‘impressions’ of sedation are more variable and imply that the ‘objective’ sedation associated with some AHs is not always fully realised.

Sedation, i.e. CNS depression, causes detrimental effects on cognitive function and psychomotor performance, and, therefore, non-sedating AHs should be selected as the first choice of treatment, especially for ambulant patients performing everyday activities in the home, at work, or on the road.

## Supporting Information

S1 Table **List of studies and test included in this review.** † The code of all subjective tests is “I”.
(DOC)

S1 Checklist **PRISMA checklist.**
(DOC)
