# Supplementary material for: Central Nervous System Effects of the Second-Generation Antihistamines Marketed in Japan -Review of Inter-Drug Differences Using the Proportional Impairment Ratio (PIR)-
Source: PLoS One. 2014 Dec 12;9(12):e114336. doi: 10.1371/journal.pone.0114336 (PMC4264760; doi:10.1371/journal.pone.0114336)
Supplement: S1 Table — List of studies and test included in this review. † The code of all subjective tests is “I”. (DOC) [file pone.0114336.s001.doc]

**Table S1. List of studies and test included in this review**

| Drug | Dose (mg) | No. of Tests showing impairment | | No. of Tests showing no impairment | |  |
| --- | --- | --- | --- | --- | --- | --- |
|  |  | Objective | Subjective † | Objective | Subjective † | References |
| cetirizine | 2.5 |  |  | 1B, 1D | 1I | Shamsi et al., 2001 [16] |
| cetirizine | 5 |  |  | 2A, 1D | 2I | Gengo et al., 1990 [17] |
| cetirizine | 5 |  |  | 1A |  | Gengo and Gabos, 1987 [18] |
| cetirizine | 5 | 1C | 1I | 1F, 1H | 1I | Nicholson and Turner, 1998 [19] |
| cetirizine | 5 |  |  | 1B, 1D | 1I | Shamsi et al., 2001 [16] |
| cetirizine | 10 |  |  | 2D | 1I | Gengo et al., 1987 [20] |
| cetirizine | 10 |  |  | 2A, 1D | 2I | Gengo et al., 1990 [17] |
| cetirizine | 10 |  |  | 1A, 2D | 2I | Gengo and Gabos, 1987 [18] |
| cetirizine | 10 |  |  | 1B, 1C, 1D | 1I | Hindmarch et al., 2001 [21] |
| cetirizine | 10 |  |  |  | 4I | Levander et al., 1991 [22] |
| cetirizine | 10 | 1H | 1I | 1C, 1F | 1I | Nicholson and Turner, 1998 [19] |
| cetirizine | 10 |  |  | 1H |  | Poza et al., 2007 [23] |
| cetirizine | 10 |  |  | 2A, 1H | 3I | Schweitzer et al., 1994 [24] |
| cetirizine | 10 |  |  | 1B, 1D | 1I | Shamsi et al., 2001 [16] |
| cetirizine | 10 |  |  | 1H | 1I | Simons et al., 1995 [25] |
| cetirizine | 10 |  | 1I | 1H |  | Simons et al., 1996 [26] |
| cetirizine | 10 |  |  |  | 1I | Tashiro et al., 2009 [27] |
| cetirizine | 10 |  |  | 1A |  | Theunissen et al., 2006 [28] |
| cetirizine | 10 |  |  | 1A | 1I | Theunissen et al., 2004 [29] |
| cetirizine | 10 |  |  | 2A, 1D, 1E |  | Volkerts et al., 1992 [30] |
| cetirizine | 10 |  |  | 1A | 2I | Walsh et al., 1992 [31] |
| cetirizine | 15 | 1C | 1I | 1F, 1H | 1I | Nicholson and Turner, 1998 [19] |
| cetirizine | 20 |  |  | 2D | 1I | Gengo et al., 1987 [20] |
| cetirizine | 20 | 1D |  | 2A | 2I | Gengo et al., 1990 [17] |
| cetirizine | 20 |  |  | 1A, 2D | 1I | Gengo and Gabos, 1987 [18] |
| cetirizine | 20 |  |  |  | 1I | Tashiro et al., 2009 [27] |
| cetirizine | 20 | 1B |  | 1B, 1F | 1I | Tashiro et al., 2004 [32] |
| desloratadine | 5 | 1E |  | 1B, 1C, 1D, 2E, 1F, 1H | 2I | Nicholson et al., 2003 [33] |
| desloratadine | 5 |  |  | 2C, 2D | 1I | Valk et al., 2004 [34] |
| desloratadine | 5 |  |  | 1A, 1F | 1I | Vuurman et al., 2004 [35] |
| ebastine | 10 |  |  | 1A |  | Brookhuis et al., 1993 [36] |
| ebastine | 10 |  |  | 2A, 1E | 1I | Hindmarch and Shamsi, 2001 [37] |
| ebastine | 10 |  |  | 2C, 1D, 1H | 1I | Hopes et al., 1992 [38] |
| ebastine | 10 |  |  | 1B, 1F | 1I | Tagawa et al., 2002 [39] |
| ebastine | 20 |  |  | 1A |  | Brookhuis et al., 1993 [36] |
| ebastine | 20 |  |  | 2A, 1E | 1I | Hindmarch and Shamsi, 2001 [37] |
| ebastine | 20 |  |  | 2C, 1D, 1H | 1I | Hopes et al., 1992 [38] |
| ebastine | 30 |  |  | 1A |  | Brookhuis et al., 1993 [36] |
| ebastine | 30 |  | 1I | 2A, 1E |  | Hindmarch and Shamsi, 2001 [37] |
| fexofenadine | 60 |  |  | 1B, 2C, 2D | 1I | Kamei et al., 2012 [40] |
| fexofenadine | 60 |  |  | 1B, 2C, 2D | 1I | Ridout and Hindmarch, 2003 [41] |
| fexofenadine | 80 |  |  | 1B, 1D, 1H | 1I | Hindmarch et al., 1999 [42] |
| fexofenadine | 120 |  |  | 1B, 1D, 1H | 1I | Hindmarch et al., 1999 [42] |
| fexofenadine | 120 |  |  | 1C, 1H |  | Kamei et al., 2003 [43] |
| fexofenadine | 120 |  |  | 1C, 1D, 1F, 1H | 2I | Nicholson et al., 2000 [44] |
| fexofenadine | 120 |  |  | 1B, 2C, 2D | 1I | Ridout and Hindmarch, 2003 [41] |
| fexofenadine | 120 |  |  | 2B, 1F | 1I | Tashiro et al., 2004 [32] |
| fexofenadine | 120 |  |  | 2A | 2I | Tashiro et al., 2005 [45] |
| fexofenadine | 180 |  |  | 1D, 3F | 1I | Bower et al., 2003 [46] |
| fexofenadine | 180 |  |  | 1B, 1D, 1H | 1I | Hindmarch et al., 1999 [42] |
| fexofenadine | 180 |  |  | 1F | 1I | Mansfield et al., 2003 [47] |
| fexofenadine | 180 |  |  | 1C, 1D, 1F, 1H | 2I | Nicholson et al., 2000 [44] |
| fexofenadine | 180 |  |  | 1B, 1D | 1I | Ridout et al., 2003 [48] |
| fexofenadine | 180 |  |  | 6F |  | Vacchiano et al., 2008 [49] |
| fexofenadine | 240 |  |  | 1C, 1D, 1F, 1H | 2I | Nicholson et al., 2000 [44] |
| fexofenadine | 360 |  |  | 1B, 2C, 1D | 1I | Hindmarch et al., 2002 [50] |
| fexofenadine | 360 |  |  | 1B, 2C, 1D, 1H | 1I | Shamsi and Hindmarch, 2000 [51] |
| levocetirizine | 5 |  |  | 1D, 1H | 1I | Gandon and Allain, 2002 [52] |
| levocetirizine | 5 |  |  | 1B, 1C, 1D | 1I | Hindmarch et al., 2001 [21] |
| levocetirizine | 5 |  |  | 2C, 2F | 1I | Verster et al., 2003 [53] |
| levocetirizine | 5 |  |  | 1A | 2I | Verster et al., 2003 [54] |
| loratadine | 10 |  |  | 1C, 1D, 1E, 2F | 1I | Bradley and Nicholson, 1987 [55] |
| loratadine | 10 | 1B |  |  |  | Gaillard et al., 1988 [56] |
| loratadine | 10 |  |  | 1B, 1D, 1H | 1I | Hindmarch et al., 1999 [42] |
| loratadine | 10 |  |  | 1B, 1C, 1D | 1I | Hindmarch et al., 2001 [21] |
| loratadine | 10 | 1B |  |  | 2I | Kavanagh et al., 2012 [57] |
| loratadine | 10 |  |  | 1A |  | O'Hanlon, 1988 [58] |
| loratadine | 10 |  |  | 1D, 1H | 1I | Roth et al., 1987 [59] |
| loratadine | 10 |  |  | 1B, 1D | 1I | Shamsi et al., 2001 [16] |
| loratadine | 10 |  |  | 1H | 1I | Simons et al., 1996 [26] |
| loratadine | 20 |  |  | 1C, 1D, 1E, 2F | 1I | Bradley and Nicholson, 1987 [55] |
| loratadine | 20 |  |  | 1A |  | O'Hanlon, 1988 [58] |
| loratadine | 20 |  |  | 1B, 1D | 1I | Shamsi et al., 2001 [16] |
| loratadine | 40 | 1D, 2F |  | 1C, 1E | 1I | Bradley and Nicholson, 1987 [55] |
| loratadine | 40 | 1H |  | 1D | 1I | Roth et al., 1987 [59] |
| loratadine | 40 |  |  | 1B, 1D | 1I | Shamsi et al., 2001 [16] |
| mequitazine | 5 | 1C |  | 2D, 1F |  | Nicholson and Stone, 1983 [60] |
| mequitazine | 5 |  |  | 1A | 1I | Theunissen et al., 2004 [29] |
| mequitazine | 10 | 1C, 1D |  | 1D, 1F |  | Nicholson and Stone, 1983 [60] |
| mequitazine | 10 | 1A |  |  |  | Theunissen et al., 2006 [28] |
| mequitazine | 10 |  |  | 1A | 1I | Theunissen et al., 2004 [29] |
| mequitazine | 15 |  |  | 1A | 1I | Theunissen et al., 2004 [29] |
| olopatadine | 5 |  |  | 1B, 2C, 2D | 1I | Kamei et al., 2012 [40] |
| olopatadine | 10 | 1H |  | 1C |  | Kamei et al., 2003 [43] |
